# TFAP2B overexpression contributes to tumor growth and a poor prognosis of human lung adenocarcinoma through modulation of ERK and VEGF/PEDF signaling

**DOI:** 10.1186/1476-4598-13-89

**Published:** 2014-04-26

**Authors:** Lingyi Fu, Ke Shi, Jingshu Wang, Wangbing Chen, Dingbo Shi, Yun Tian, Wei Guo, Wendan Yu, Xiangsheng Xiao, Tiebang Kang, Shusen Wang, Wenlin Huang, Wuguo Deng

**Affiliations:** 1 State Key Laboratory of Oncology in South China, Colaborative Innovation Center of Cancer Medicine, Sun Yat-sen University Cancer Center, 651 Dongfeng East Road, Guangzhou 510060, China; 2 Department of Geratology, Xiangya Hospital, Central South University, Changsha, China; 3 Institute of Cancer Stem Cell, Dalian Medical University, Dalian, China; 4 State Key Laboratory of Targeted Drug for Tumors of Guangdong Province, Guangzhou Double Bioproduct Inc., Guangzhou, China

**Keywords:** Lung cancer, TFAP2B, ERK, VEGF, Caspase

## Abstract

**Background:**

TFAP2B is a member of the AP2 transcription factor family, which orchestrates a variety of cell processes. However, the roles of TFAP2B in regulating carcinogenesis remain largely unknown. Here, we investigated the regulatory effects of TFAP2B on lung adenocarcinomas growth and identified the underlying mechanisms of actions in non-small cell lung cancer (NSCLC) cells.

**Methods:**

We first examined the expression of TFAP2B in lung cancer cell lines and tumor tissues. We also analyzed the prognostic predicting value of TFAP2B in lung adenocarcinomas. Then we investigated the molecular mechanisms by which TFAP2B knockdown or overexpression regulated lung cancer cell growth, angiogenesis and apoptosis, and further confirmed the role of TFAP2B in tumor growth in a lung cancer xenograft mouse model.

**Results:**

TFAP2B was highly expressed in NSCLC cell lines and tumor tissues. Strong TFAP2B expression showed a positive correlation with the poor prognoses of patients with lung adenocarcinomas (P < 0.001). TFAP2B knockdown by siRNA significantly inhibited cell growth and induced apoptosis in NSCLC cells in vitro and in a lung cancer subcutaneous xenograft model, whereas TFAP2B overexpression promoted cell growth. The observed regulation of cell growth was accompanied by the TFAP2B-mediated modulation of the ERK/p38, caspase/cytochrome-c and VEGF/PEDF-dependent signaling pathways in NSCLC cells.

**Conclusions:**

These results indicate that TFAP2B plays a critical role in regulating lung adenocarcinomas growth and could serve as a promising therapeutic target for lung cancer treatment.

## Introduction

Lung cancer is the leading cause of cancer-related deaths worldwide [1,2]. Non-small-cell lung cancer (NSCLC) is a major form of lung cancer, and chemotherapy and surgical resection are the main therapeutic strategies [3-5]. Unfortunately, the prognosis of patients with lung cancer remains poor even after curative surgery due to the high incidence of tumor recurrence and distant metastases [6,7]. In recent years, therapies selectively targeting cell signaling pathways, such as VEGF, EGFR, KRAS, BRAF, ALK, HER2, MET, TITF-1, p53, and LKB1, have both provided a better understanding of NSCLC and have been used as prognostic factors or targets for individualized therapy [8]. Therefore, there is an urgent need for a further understanding of the molecular mechanisms in lung carcinogenesis and for the identification of effective prognostic and diagnostic biomarkers and new therapeutic targets.

TFAP2B (transcription factor activating enhancer-binding protein 2B) is a member of the AP-2 transcription factor family, which consists of five different yet closely related 50-kDa isoforms [9-12]. The TFAP2 factors present a conserved helix-span-helix dimerization domain preceded by a DNA-binding and a transactivation domain [13,14]. By binding to GC-rich consensus sequences, TFAP-2 factors regulate the expression of many downstream genes, thereby orchestrating a variety of cell processes, particularly cell induction, differentiation, survival, and proliferation and apoptosis within various developmental contexts [15-19]. TFAP2A and TFAP2C have been shown to participate in tumorigenesis by controlling the expression of many cancer-related genes, such as vascular endothelial growth factor (VEGF), P21, Rb, TP53, ERa, BCL2, cKIT, MMP-2, E-cadherin, and c-myc [20-24]. Although genetic variations of TFAP2B are associated with adipocytokine regulation and type 2 diabetes mellitus [25,26], the role of TFAP2B in regulating cancer-related gene expression remains largely unknown.

In the present study, we examined the expression of TFAP2B at protein levels in lung cancer cell lines and tumor tissues, evaluated the effect of TFAP2B knockdown or overexpression on lung cancer cell growth, and further elucidated the underlying molecular mechanisms. We also analyzed lung adenocarcinoma specimens in a tissue array and evaluated the prognostic predicting value of TFAP2B in lung adenocarcinomas. The role of TFAP2B in lung cancer growth was further confirmed *in vivo* using a lung cancer xenograft model. Our findings provide new insight into the understanding of the biological role of TFAP2B in lung cancer and suggest that TFAP2B could serve as a novel therapeutic target for lung cancer treatment.

## Materials and methods

### Cell lines and cell culture

Human lung cancer cell lines (H1299, A549, H460) and normal cell lines (293, HBE and fibroblast) were obtained from the American Type Culture Collection (ATCC, Manassas, VA). The cells were cultured as monolayers in RPMI-1640 culture medium (Invitrogen, Carlsbad, CA) supplemented with 10% fetal bovine serum, 100 μg/ml penicillin, and 100 μg/ml streptomycin and maintained in an incubator with a humidified atmosphere of 95% air and 5% CO_2_ at 37°C.

### Reagents and antibodies

Antibodies against TFAP2B, GAPDH, VEGF, PEDF, were obtained from Santa Cruz Biotechnology (Santa Cruz, CA). Antibodies against cytochrome-c, PARP, caspase-3/8/9, BAX, and Bcl-2 were purchased from Cell Signaling (Beverly, MA).

### Tissue array and immunohistochemistry

The tissue array consisted of 147 formalin-fixed, paraffin-embedded (FFPE) lung adenocarcinomas and corresponding adjacent normal tissues. These tissue samples were previously obtained with informed consent from patients having no anticancer treatment prior to tumor resection. The tissue specimens were histologically examined and classified according to the 2004 World Health Organization classification system [27]. Detailed clinical and pathologic information, including the clinical and pathologic tumor-node-metastasis (TNM) stage, overall survival (OS) duration, and time to recurrence, was available for all cases. The pathological TNM status of all of the lung adenocarcinomas was assessed according to the criteria of the seventh edition of the American Joint Committee on Cancer (2010).

Immunohistochemistry was conducted using Envisionþ Kit/HRP (DakoCytomation). Briefly, slides were immersed in Target Retrieval Solution (pH 9; DakoCytomation) and boiled at 108°C for 15 min in an autoclave for antigen retrieval. The anti-TFAP2B antibody was added to each slide after blocking of the endogenous peroxidase and proteins, and the sections were incubated with HRP-labeled anti-rabbit IgG as the secondary antibody. The substrate-chromogen was added, and the specimens were counterstained with hematoxylin. A negative control was obtained by replacing the primary antibody with normal rabbit IgG.

To evaluate the immunohistochemical staining, two independent observers blinded to the clinicopathologic information performed scoring using light microscopy (magnification 20×). The intensity of TFAP2B staining was semiquantitatively evaluated using the following criteria: strongly positive (scored 2+), dark-brown staining in more than 50% of the tumor cells, completely obscuring the nucleus; weakly positive (scored 1+), any lesser degree of brown staining appreciable in the tumor cell nucleus; absent (scored 0), no appreciable staining in the tumor cells. Cases were accepted as strongly positive if 2 or more investigators independently defined them as such.

### Western blot analysis

The proteins in cell lysates were separated by 10% sodium dodecyl sulfate-polyacrylamide gel electrophoresis (SDS-PAGE) (Bio-Rad, Hercules, CA) and electrophoretically transferred to a PVDF membrane (Amersham Pharmacia Biotech, Piscataway, NJ). The western blots were probed with specific antibodies, and the protein bands were detected using enhanced chemiluminescence.

### Preparation of siRNA or plasmid DC nanoparticles

The TFAP2B siRNA and overexpression plasmid were purchased from Shanghai GenePharma Co. (Shanghai China). The sequence of the human TFAP2B-specific siRNA was 5′-GGA CCA GUC UGU CAU UAA ATT-3′ and 5′-CCC GAA AGA AUA UGC UGU UTT-3′, and the scramble siRNA was 5′-UUC UCC GAA CGU GUC ACG UTT-3′. The siRNA and plasmid were incorporated with additional chemical modifications for superior serum stability in the *in vivo* applications, and the knockdown efficiency was validated *in vitro*[28,29]. For the *in vivo* delivery of the siRNA and plasmid into tumors, the sequences were first encapsulated into DOTAP-cholesterol (DC) (Avanti Polar, Birmingham, AL, USA) nanoparticles that had validated by many analyses by dissolving in sterilized de-ionized water and then mixing with the nanosomes.

### Transient transfection

A total of 2 × 10^5^ H1299 cells were seeded into each well of a six-well tissue culture plate (Costar). The next day (when the cells were 70-80% confluent), the culture medium was aspirated, and the cell monolayer was washed with prewarmed sterile phosphate-buffered saline (PBS). The cells were transfected with the siRNA or plasmid at the indicated dose using the DC nanoparticles. The cells were harvested after 48 h of transfection, and western blot analyses or other experiments were performed.

### Cell viability assay

A cell proliferation assay was performed using the MTT assay (Roche Diagnosis, Indianapolis, IN). Briefly, cells were plated in 96-well plates (2000 cells/well) in triplicate and treated with siRNA or plasmid. Cell viability was determined after 48 h.

### Anchorage-independent colony formation

Cells were transfected with TFAP2B siRNA or plasmid for 24 h, trypsinized, and resuspended as single cells. The cells (8 × 10^3^/ml) were then mixed in 1 ml of 0.3% McCoy’s 5a agar containing 10% FBS. The cultures were maintained in a 37°C/5% CO_2_ incubator for 14 days. The cell clones were then washed three times with phosphate-buffered saline (PBS), fixed in methanol for 10 minutes, and stained with Crystal Violet for 10 minutes at room temperature. The dye was washed off, and the colonies that contained more than 50 cells were counted.

### Determination of VEGF and PEDF production by ELISA

H1299 cells were seeded in 96-well plates and treated with TFAP2B siRNA at 100 nM for 48 hours. The VEGF and PEDF levels in the culture media were quantified using a VEGF Immunoassay Kit (968962) (R & D Systems, Minneapolis, MN) and a Chemikine PEDF ELISA Kit (CYT420) (Chemikine, Billerica, MA) according to the manufacturer’s protocols.

### Apoptosis assay

H1299 cells were transfected with TFAP2B siRNA. At 48 h after transfection, the cells were harvested by trypsinization and fixed in 70% cold ethanol for 30 minutes, then stained with 5 μl Annexin V-FITC and 5 μl PI (propidium iodide) using an Annexin V-FITC/PI-staining kit (Becton Dickinson, CA, USA). The cells were placed at room temperature for 15 min in the dark and then analyzed using flow cytometry (EPICS XL; Beckman Coulter). Apoptosis was calculated in terms of the FITC-positive cells, and the PI staining was used to perform a cell cycle analysis. The raw data were analyzed using Multicycle for Windows (Beckman Coulter).

### Immunofluorescence and confocal microscopy

Cells were seeded in two-well chamber slides at a density of 1 × 10^5^ cells per well. After 48 h, the cells were washed with PBS, fixed with 4% paraformaldehyde solution, and permeabilized with 0.1% Triton X-100. The cells were incubated with a rabbit anti-cytochrome-c antibody and then incubated with a rhodamine-conjugated goat anti-rabbit IgG (Santa Cruz Biotechnology). The nuclei were stained with 4′, 6-diamidino-2-phenylindole (DAPI), and the cells were examined under a fluorescence microscope.

### Tumor growth inhibition by TFAP2B shRNA in a xenograft mouse model

Endothelial-cell tube-formation assay 200 μL of growth factor-reduced Matrigel (BD Biosciences, USA) was pipetted into each well of a 24-well plate and polymerized for 30 min at 37°C. Human umbilical vein endothelial cells were transfected with si-TFAP2B for 24 h, then harvested by trypsin treatment and suspended in conditioned medium. Then 2 × 10^4^ human umbilical vein endothelial cells in 300 μL conditioned medium were added to each well and incubated at 37°C, 5% CO_2_ for 8 h. The cultures were photographed by microscopy.

To determine the effect of TFAP2B shRNA on lung cancer cell growth in a xenograft model, A549 cells (2 × 10^6^) were inoculated subcutaneously into the flank of nude mice. Once palpable tumors were observed, tumor volume measurements were obtained every 3 days using calipers. The tumor volume was calculated using the following formula: V = (width^2^ × length)/2. The body weights were also recorded. At two weeks after injection, the mice were randomized into 2 groups (5 mice/group). Group 1 received an injection of In Vivo Ready nonspecific shRNA, and group 2 received an injection of In Vivo Ready TFAP2B shRNA. The DC nanoparticle-encapsulated shRNA duplexes were injected into the tumors using insulin syringes at a concentration of 10 μg of shRNA/50 mm^3^ of tumor volume [30]. The two groups were treated twice per week for three weeks. Upon termination, the tumors were harvested and weighted. The animal experiments were approved by the Animal Research Committee of Sun Yat-sen University Cancer Center and were performed in accordance with established guidelines.

### Evaluation of angiogenesis factors in xenograft tumor tissues

The tumor tissues from the above treated animals were collected and soaked in 10% formalin and then embedded in paraffin for the analysis. The sections were stained with H&E according to standard immunohistochemical procedures. To assess the impact of the TFAP2B shRNA on angiogenesis factors *in vivo*, the embedded tissues were stained for VEGF and PEDF to investigate angiogenic factor expression. A negative control was obtained by replacing the primary antibody with normal rabbit or mouse IgG. The immunoreactive-positive cells from each of the differently treated tumor tissue sections were measured at 200× magnification using a light microscope. The amount of protein was analyzed by the integral optical density (IOD) using IPP (Image Plus Pro 6.0, Bethesda, MD, USA).The images were examined under a Nikon TC200 fluorescence microscope equipped with a digital camera.

### Statistical analysis

A statistical analysis was performed using the SPSS statistical software package (standard version 16.0; SPSS, Chicago, IL). Strong TFAP2B immunoreactivity was assessed for the association with clinicopathologic variables, such as gender, age, and pathologic tumor-node-metastasis stage using the Pearson Chi-square test. Survival curves were calculated from the date of surgery to the time of death related to NSCLC or to the last follow-up observation. Kaplan–Meier curves were calculated for each relevant variable and for TFAP2B expression; the differences in survival time among the patient subgroups were analyzed by the log-rank test. Univariate and multivariate analyses were performed with the Cox proportional hazard regression model to determine associations between the clinicopathologic variables and cancer-related mortality. First, we analyzed the associations between death and possible prognostic factors, including TFAP2B expression, gender, age, pT classification, and pN classification, taking into consideration one factor at a time. Second, a multivariate analysis was applied using forward (stepwise) procedures.

## Results

### TFAP2B is highly expressed in lung cancer and associated with a poor prognosis of lung adenocarcinomas patients

**Figure 1:**
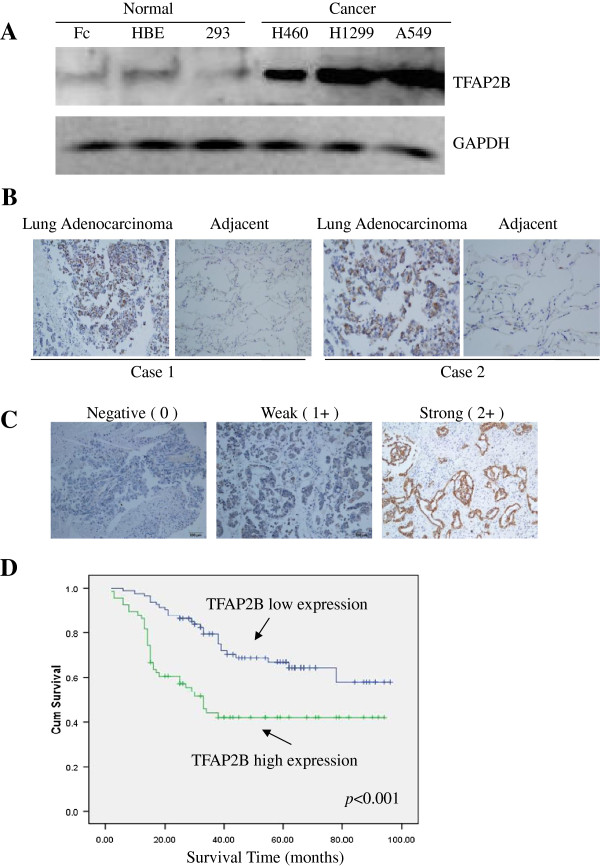
**TFAP2B protein is highly expressed in lung cancer cells and tumor tissues and associated with a poor prognosis.** The expression of TFAP2B proteins in various lung cancer cell lines and human normal cells **(A)**, as well as in tumor tissues **(B)** were analyzed by Western blotting and immunohistochemical analyses using a human TFAP2B antibody. Original magnification, 200×. **(C)** Immunohistochemical staining of a tissue microarray with lung adenocarcinoma sections. The representative areas of lung adenocarcinomas stained using a monoclonal anti-human TFAP2B antibody and scored as 0, 1+, and 2+. A strong positive staining was observed in cancer cells, whereas the adjacent area staining was weak. Original magnification, 200×. **(D)** TFAP2B overexpression correlates with a poor prognosis in patients with lung adenocarcinomas. Kaplan-Meier curves of overall survival in all (n = 147) patients.

We first detected the expression of TFAP2B at protein levels in human normal cell lines (fibroblasts, HBE, 293) and lung cancer cell lines (H460, H1299, A549) by Western blotting (Figure 1A). Among the cell lines examined, the lung cancer cell lines expressed high levels of TFAP2B protein, though the expression of TFAP2B was faint in normal human lung cell lines. We also immunohistochemically detected the expression of the TFAP2B protein in lung adenocarcinoma and adjacent area. As shown in Figure 1B, positive staining of TFAP2B was observed in lung adenocarcinoma tissue but not in adjacent normal lung cells, suggesting that TFAP2B might be a potential biomarker of lung cancer.

We evaluated the expression of TFAP2B using TMA-containing tumors from 147 patients with lung adenocarcinoma with full clinical annotation to assess the biological and clinicopathologic significance. An immunohistochemistry analysis revealed that TFAP2B was abundantly accumulated in the cytoplasm of lung cancer cells. We classified expression on the tissue array at three levels: strongly positive (2+ score), weakly positive (1+ score), and absent (0 score) (Figure 1C). The disease-free survival curves revealed an unfavorable prognosis for the high-TFAP2B group compared to the low-TFAP2B group (*P* < 0.001, log-rank test; Figure 1D).

There was no statistically significant relationship between TFAP2B expression and gender (*P* = 0.322), age (*P* = 0.141), pT stage (*P* = 0.512), and pN stage (*P* = 0.060) (Additional file 1. Table S1). We also used a univariate analysis to evaluate associations between the patient prognosis and several clinicopathologic factors, including TFAP2B expression (score of 2+ vs. 0, 1+), gender (male vs. female), age (≥ 60 vs. < 60 years), pT stage (tumor size, T3-T4 vs. T1-T2), and pN stage (lymph node metastasis, N1–N2-N3–N4 vs. N0). All of these parameters except gender and age were significantly associated with a poor prognosis. Multivariate Cox proportional hazards regression analyses showed that strong TFAP2B positivity and pN stage were independent prognostic factors for NSCLC: Hazard ratio (HR), 2.349; 95% CI, 1.411-3.910; *P* = 0.001. (Additional file 1: Table S2).

### TFAP2B regulates cell proliferation

**Figure 2:**
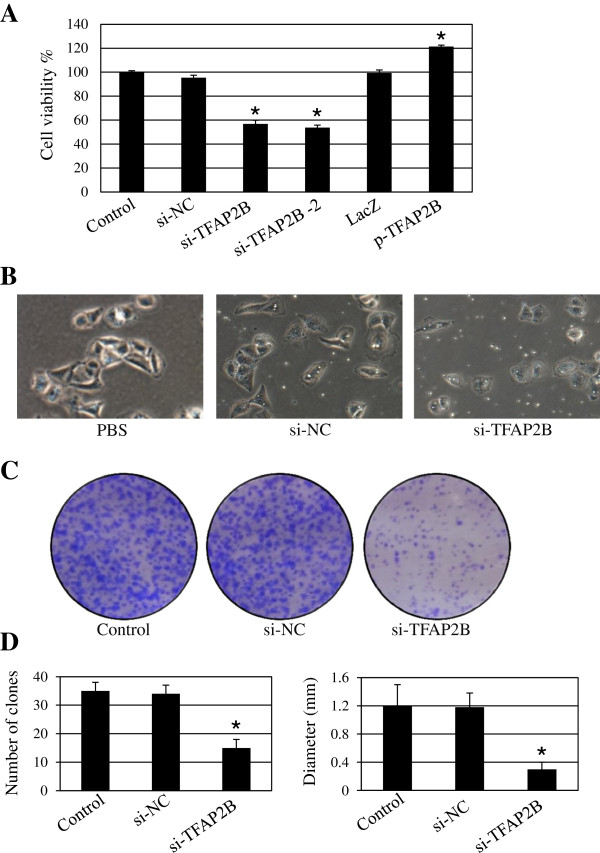
**TFAP2B regulates cell proliferation.** Human H1299 cells were transfected with two different sequences targeting TFAP2B (si-TFAP2B and si-TFAP2B-2) or an TFAP2B-overexpressing plasmid (p-TFAP2B). At 48 hours after transfection, the cell viability was determined by an MTT assay **(A),** and the cells were photographed **(B)**. Cells treated with DMSO were used as the reference group, with the cell viability set at 100%. The percent cell viability in each treatment group was calculated relative to the cells treated with the vehicle control. The tumor cell H1299-induced colony formation was also analyzed **(C),** and the colony formation rate and colony size were calculated **(D)**. The data are presented as the mean ± SD of three tests. *P < 0.05, significant differences between the treatment groups and control groups. si-NC means non-specific siRNA control.

To assess the effects of TFAP2B on cell proliferation, we quantitatively analyzed cell viability by the MTT assay. TFAP2B knockdown by transfection with two different sequences targeting TFAP2B (si-TFAP2B and si-TFAP2B-2) potently inhibited the proliferation of H1299 cells by comparison with the transfection with a non-specific scramble siRNA sequence control (si-NC) (Figure 2A), which excluded the possibility of the off-target effect of TFAP2B siRNA. Based on the consistent results of these two siRNA sequences, we selected the si-TFAP2B for our next experiments. In contrast, TFAP2B overexpression by transfection with a TFAP2B-expressing vector significantly increased cell viability compared to the control LacZ (Figure 2A).

We next analyzed the effect of TFAP2B on the changes in cell morphology. We found that cells in the control group formed a cell layer, and more spread and filopodia were observed, while TFAP2B knockdown markedly reduced cell-to-cell contact and led to a lower spreading with fewer formation of filopodia (Figure 2B).

We also performed an anchorage-independent colony formation assay to confirm the effects of TFAP2B on cancer cell growth. TFAP2B knockdown by siRNA significantly inhibited cell clonogenicity, resulting in a marked decrease in both the colony formation ratio and colony size. These results suggested that reductions in TFAP2B levels decrease the ability of lung cancer cells to form colonies in soft agar, which was consistent with the MTT results (P < 0.001, Figure 2C and D).

### TFAP2B knockdown inactivates ERK/p38 MAPK signaling

**Figure 3:**
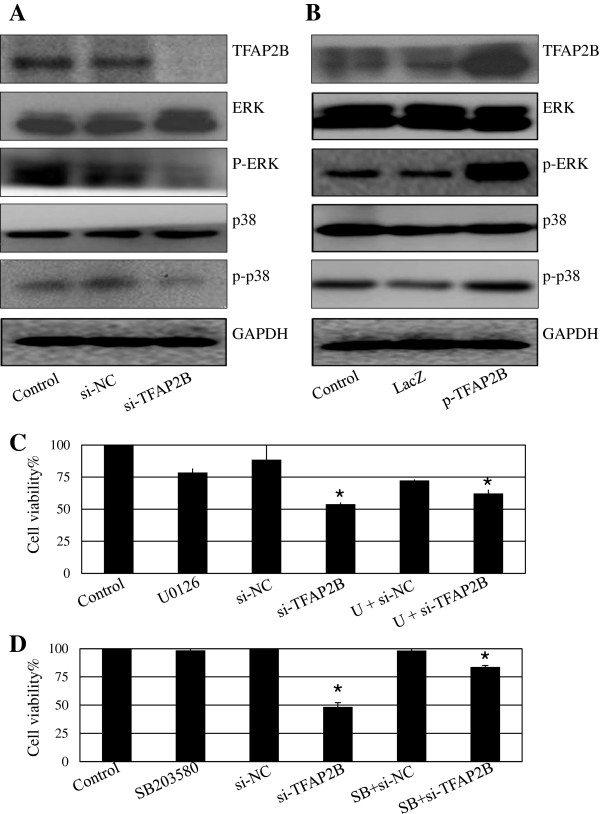
**TFAP2B knockdown inhibits ERK/p38 MAPK signaling pathway.** Human H1299 cells were transfected with the DC-based TFAP2B siRNA (si-TFAP2B) **(A)** or TFAP2B-expressing plasmids **(B)**. At 48 hours after transfection, the levels of TFAP2B, total and phosphorylated ERK and p38 proteins were analyzed by Western blotting. H1299 cells were pre-treated with an ERK inhibitor U0126 **(C)** or a p38 inhibitor SB03580 **(D)** for 4 h, then transfected the cells with TFAP2B siRNA or expressing plasimd. After 48 h, cell viability was determined by MTT analysis. *P < 0.05, significant differences between the treatment groups and DMSO. U means U0126 and SB means SB03580.

To identify the potential molecular mechanisms by which TFAP2B knockdown inhibited lung cancer cell growth and proliferation, we analyzed the activities of several pro-survival proteins by Western blot and showed that TFAP2B knockdown dramatically suppressed the phosphorylation of ERK1/2 and p38, whereas the levels of total ERK1/2 and p38 protein did not change (Figure 3A). Conversely, TFAP2B overexpression increased the phosphorylation of ERK1/2 and p38, thereby leading to an activation of the MAPK signaling pathways (Figure 3B).

To confirm the involvement of the ERK/p38 MAPK signaling pathway in the TFAP2B-mediated regulation of cell growth, we analyzed the effects of the ERK and p38-selective inhibitors (U0126 and SB03580) on TFAP2B siRNA-mediated inhibition of cell viability in lung cancer cells. Pretreatment of the cells with U0126 or SB03580 alone slightly inhibited cell viability. However, addition of TFAP2B siRNA did not significantly enhance the U0126 or SB03580-mediated inhibition of cell viability (Figure 3C,D) due to the pre-blockage of the ERK/p38 signaling pathway by U0126 or SB03580. These results indicate that TFAP2B functions partially through the modulation of the ERK/p38 signaling to regulate tumor cell growth.

### TFAP2B knockdown induces apoptosis in lung cancer cells

**Figure 4:**
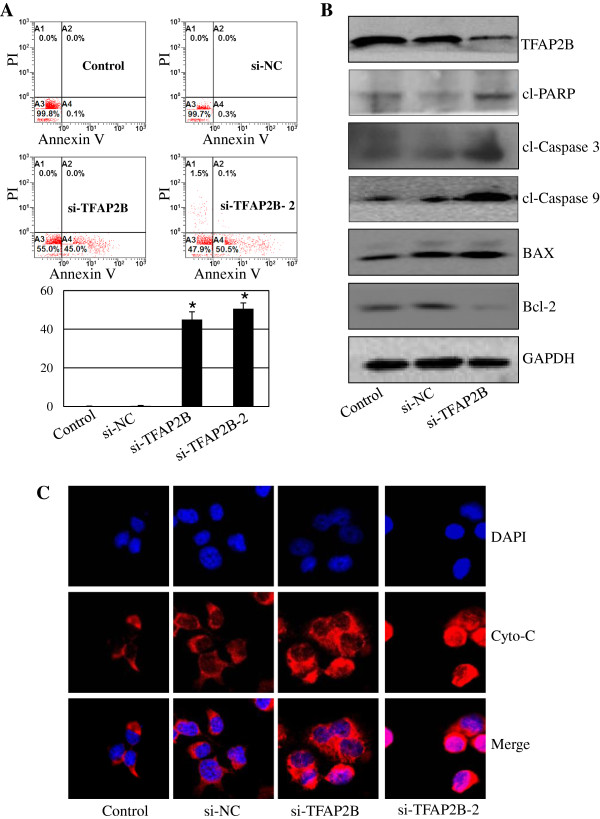
**TFAP2B knockdown activates caspase-dependent apoptotic pathway.** Human H1299 cells were transfected with two different sequences targeting TFAP2B (si-TFAP2B and si-TFAP2B-2). At 48 hours after transfection, FACS analysis was used to determine apoptosis **(A)**. The levels of cleaved PARP, cleaved caspase-3/9, BAX, and Bcl-2 proteins were analyzed by western blotting **(B)**. The release of cytochrome-c was analyzed by an immunofluorescence imaging analysis to monitor cytochrome-c release from the inter-mitochondrial space into the cytosol **(C)**. Apoptosis is represented by the relative percentage of apoptotic cells versus that of the DMSO-treated cells. *P < 0.05, significant differences between the treatment groups and control groups.

We also assessed the proportion of apoptosis in H1299 cells transfected with two different sequences targeting TFAP2B (si-TFAP2B and si-TFAP2B-2) or conrtrol siRNA (NC-siRNA) by Annexin V/PI staining-based FACS analysis. The knockdown of TFAP2B by siRNA resulted in a significant induction in H1299 cell apoptosis. Conversely, apoptosis was not observed in the cells transfected with the nonspecific siRNA (Figure 4A).

Caspase activation is an important event in the apoptosis signaling pathway. Thus, to confirm the effect of TFAP2B knockdown on apoptosis, we next detected the expression of certain pro-apoptotic and anti-apoptotic proteins, PARP, caspase-3/9, BAX, and Bcl-2 by Western blotting analysis. As shown in Figure 4B, TFAP2B knockdown markedly induced the activation of PARP and caspase-3/9, resulting in an increase in the levels of cleaved PARP and caspase-3/9 proteins. TFAP2B knockdown also increased the expression of the pro-apoptotic protein BAX and suppressed the expression of the anti-apoptotic protein BCl-2.

As cytochrome-c release is an important event in the caspase-dependent apoptosis pathway, we next performed an immunofluorescence imaging (IF) analysis to monitor changes in the subcellular localization of cytochrome-c in H1299 cells. Transfection of cells with two different sequences targeting TFAP2B (si-TFAP2B and si-TFAP2B-2) triggered the release of cytochrome-c from the inter-mitochondrial space into the cytosol by comparison with the control siRNA (si-NC) (Figure 4C). These results demonstrate that TFAP2B may control several aspects of apoptosis signaling.

### TFAP2B regulates the VEGF/PEDF ratio

**Figure 5:**
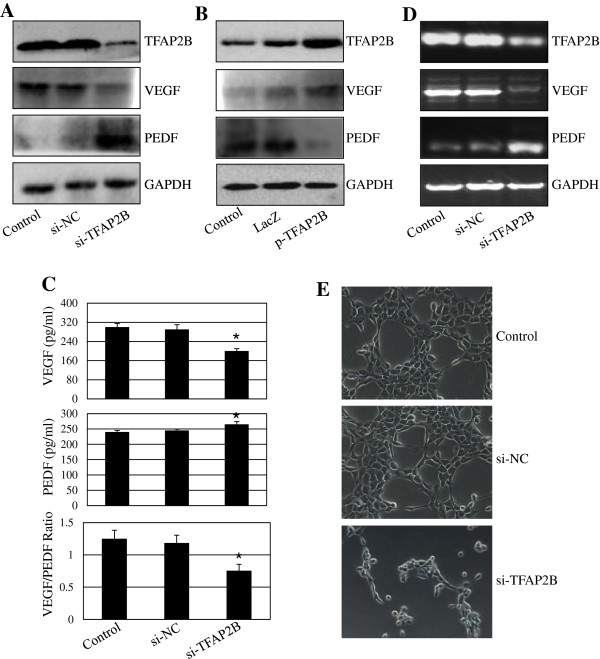
**TFAP2B knockdown inhibits VEGF/PEDF signaling and angiogenesis.** Human H1299 cells were transfected with DC-based TFAP2B siRNA (si-TFAP2B) or overexpression plasmid (p-TFAP2B) nanoparticles. At 48 hours after transfection, the effects of TFAP2B knockdown or overexpression on the expression of TFAP2B, VEGF and PEDF at protein levels **(A, B)** and mRNA levels **(D)** were detected by Western blotting and RT-PCR, respectively. The amount of VEGF and PEDF proteins released in cell to the culture media were determined by ELISA, and the ratio of VEGF to PEDF in the media was also analyzed (**C)**. Human umbilical vein endothelial cells (HUVEC) were transfected with DC-based TFAP2B siRNA (si-TFAP2B) nanoparticles. At 72 hours after transfection, the effect of TFAP2B knockdown on tube formation assay was analyzed **(E)**. The data are presented as the mean ± SD of three separate experiments. *P < 0.05, significant differences between the treatment groups and control groups.

VEGF/PEDF-mediated angiogenesis plays an important role in tumor growth. We next determined the effect of TFAP2B on the expression of VEGF and PEDF proteins. Transfection with TFAP2B siRNA dramatically inhibited the expression of TFAP2B and VEGF proteins and increased the expression of PEDF protein (Figure 5A), resulting in a significant reduction in the VEGF/PEDF ratio; in contrast, TFAP2B overexpression showed an opposite effect (Figure 5B). We also examined the release of VEGF and PEDF proteins into the cell culture media by ELISA. TFAP2B knockdown by siRNA significantly inhibited the release of VEGF protein but activated the release of PEDF (Figure 5C). Moreover, we determined the effect of TFAP2B on the expressions of VEGF and PEDF at transcriptional levels. Knockdown of TFAP2B by siRNA transcriptionally repressed TFAP2B and VEGF expression and activated PEDF expression at mRNA levels (Figure 5D).

We next tested the effect of TFAP2B knockdown on angiogenesis by analyzing the tube formation ability of human umbilical vein endothelial cells (HUVEC). The results showed that transfection with TFAP2B siRNA markedly inhibited tube formation ability of HUVEC as compared with the control siRNA (si-NC) (Figure 5E).

### TFAP2B inhibits tumor growth and angiogenesis *in vivo*

**Figure 6:**
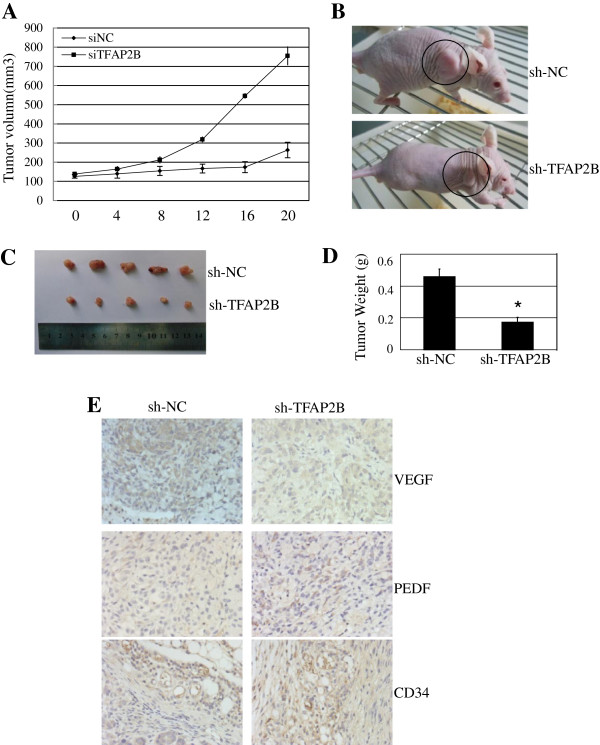
**The knockdown of TFAP2B inhibits tumor growth *in vivo*.** DC nanoparticle-encapsulated TFAP2B shRNA (sh-TFAP2B) or non-specific scramble shRNA (sh-NC) was injected into the tumor region. Day 0 corresponds to 2 weeks after inoculation of A549 cells and the first treatment when the mean tumor volume was 52 mm^3^. The tumor diameters were measured at a regular interval of 4 days for up to 22 days with a digital caliper, and the tumor volume was calculated **(A)**. Tumor xenografts in the mice were photographed **(B)**. Xenografts were harvested at 22 days after treatment. Pictures of the tumors were obtained **(C)**, and the weights of the tumors were analyzed **(D)**. The expression levels of VEGF, PEDF and CD34 proteins in tumor tissues were detected by immunohistochemical staining **(E)**. The data are presented as the mean ± SD of three tests. *P* < 0.05, significant differences between the TFAP2B shRNA groups and non-specific scramble shRNA groups. n = 7 mice/group. Magnification, 200 ×.

To further confirm the significant association of TFAP2B expression with lung cancer cell survival and clinical outcome, we verified the essential role of TFAP2B in regulating lung cancer growth *in vivo*. A549 cells were injected subcutaneously into the flank of nude mice, and visible tumors developed at the injection sites after 2 weeks (mean tumor volume = 52 mm^3^). The DC nanoparticle-encapsulated TFAP2B shRNA was then intratumorally injected twice per week for three weeks. Knockdown of TFAP2B by shRNA significantly suppressed tumor growth (Figure 6A and B) and tumor weight (Figure 6C and D) compared to the nonspecific shRNA treatment. We also analyzed the levels of TFAP2B expression in the tumors by immunohistochemical staining and showed a significant decrease in TFAP2B expression in the tumors treated with TFAP2B shRNA (data not shown).

To elucidate the potential mechanisms involved in tumor growth inhibition by TFAP2BshRNA *in vivo*, we also analyzed the expression of VEGF, PEDF and micro-vessel density marker CD34 in tumors by immunohistochemical staining and found that VEGF expression was inhibited and the mean MVD was reduced (Figure 6E). These *in vivo* results were consistent with those observed *in vitro* and indicated that TFAP2B could be a useful therapeutic target for lung cancer.

## Discussion

In this study, we evaluated the biological role and clinical significance of TFAP2B in lung cancer carcinogenesis. TFAP2B belongs to the TFAP2 family. TFAP2A and TFAP2C have been implicated in cancer progression, vascularization, metastasis, and recurrence [31-33]. However, the biological roles and clinical significance of TFAP2B and its precise molecular mechanisms in lung cancer have not been reported.

We demonstrated the high expression of TFAP2B in lung cancer cells, tumor tissues, and lung adenocarcinoma samples compared to normal cells and normal human organ tissues. To evaluate our hypothesis that TFAP2B plays a potential oncogenic role in lung cancer, we performed *in vitro* studies to investigate the underlying molecular mechanisms and found that TFAP2B knockdown inhibited cell viability, clonogenicity, and angiogenesis and induced apoptosis *in vitro* but that TFAP2B overexpression had the opposite effect in H1299 lung cancer cells. We also demonstrated the TFAP2B-mediated regulation of tumor growth in a A549 lung cancer xenograft mouse model *in vivo*. In the *in vitro* experiment, we performed all the experiments in H1299 cells while in vivo experiments we used A549 cells because of the fact that H1299 cells are easily transfected and A549 cells has a higher ability to form xenograft in nude mice.

The clinicopathologic data from our tissue array showed that patients with lung adenocarcinomas, which highly expressed the TFAP2B protein, had shorter survival periods than patients with TFAP2B-weakly positive/negative tumors. Moreover, a multivariate analysis showed that strong TFAP2B positivity is an independent prognostic factor for a poor outcome. These findings suggest a potential oncogenic role of TFAP2B in human lung cancer.

Apoptosis has been demonstrated to represent a protective mechanism against neoplastic transformation and plays a crucial role in the response of cancer to chemotherapy and radiation therapy [34-37]. In this study, we showed that the induction of apoptosis in human lung cancer cells by TFAP2B knockdown was mediated by cytochrome-c and caspase-dependent apoptosis pathways. We found that TFAP2B siRNA induced the activation of caspase proteins and promoted the release of cytochrome-c from the mitochondria to cytosol. Our results therefore suggest that the antitumor effect of TFAP2B knockdown in lung cancer cells is associated with the increased activation of the cytochrome-c and caspase-dependent apoptotic pathway.

VEGF has been recognized as one of the principal initiators for the development and progression of vascularization, and VEGF was shown to have the most involvement both during tumor angiogenesis and also in mediating tumor cell growth and survival [38-40]. PEDF counterbalances the effect of VEGF [41,42], and an increased ratio of VEGF/PEDF is required for angiogenesis and tumor growth [43]. Our result also showed that TFAP2B knockdown in lung cancer cell lines led to a significant reduction in the VEGF/PEDF ratio at the mRNA and protein levels, and we also detected a physical interaction between the VEGF with TFAP2B proteins, suggesting that TFAP2B at least partially targets VEGF/PEDF signaling.

Our results demonstrated that TFAP2B might regulate lung cancer cell proliferation by targeting ERK/p38, VEGF/PEDF, and caspase-dependent signaling pathways. However, the detailed mechanisms by which TFAP2B simultaneously regulates these signaling pathways remain to be elucidated.

Moreover, we demonstrated that the knockdown of TFAP2B using DC-based TFAP2B shRNA nanoparticles markedly inhibited tumor growth in a lung cancer xenograft mouse model, confirming the role of TFAP2B in tumor growth and survival. Immunohistochemistry on the xenograft tumors showed that TFAP2B knockdown inhibited the angiogenesis-related protein factor VEGF. Therefore, these *in vivo* experiments confirmed the tumor-inhibition effects by TFAP2B knockdown *in vitro* and provide a rationale for the pharmacologic investigation of TFAP2B as a novel therapeutic target in lung cancer cells. Regardless, the detailed mechanisms remain to be elucidated.

In conclusion, we demonstrated that TFAP2B plays a critical role in human lung carcinogenesis by simultaneously regulating multiple signaling pathways, such as the ERK/p38, VEGF/PEDF, and caspase-dependent pathways. Our study also demonstrated that high TFAP2B expression independently predicted a worse overall survival in patients with lung adenocarcinomas. Our findings suggest that TFAP2B overexpression might help to identify NSCLC patients with a poor prognosis and could therefore serve as a potential prognostic biomarker and therapeutic target for lung cancer.

## Competing interests

The authors declare no conflict of interest.

## Authors’ contributions

Participated in research design: LF, KS, SW, WH, WD. Conducted experiments: LF, JW, WC, DS, YT, WY, WG. Performed data analysis: LF, KS, XX, TK. Wrote or contributed to the writing of the manuscript: WD, LF, KS. All authors read and approved the final manuscript.

## Supplementary Material

Additional file 1: Table S1 Association of TFAP2B expression with patient’s clinicopathological features in lung adenocarcinomas. Of the 147 lung adenocarcinoma samples, TFAP2B stained strongly in 66 cases (44.9%, 2+ score), staining weakly stained or not at all in 81 cases (55.1%, 1+ or 0 score). There was no statistically significant relationship between TFAP2B expression and gender (*P* = 0.322), age (*P* = 0.141), pT stage (*P* = 0.512), and pN stage (*P* = 0.060). **Table S2.** Cox proportional hazards model analysis of prognostic factors in patients with lung adenocarcinomas. Univariate analysis was used to evaluate associations between the patient prognosis and several clinicopathologic factors, including TFAP2B expression (score of 2+ vs. 0, 1+), gender (male vs. female), age (≥ 60 vs. < 60 years), pT stage (tumor size, T3-T4 vs. T1-T2), and pN stage (lymph node metastasis, N1–N2-N3–N4 vs. N0). All of these parameters except gender and age were significantly associated with a poor prognosis. Multivariate Cox proportional hazards regression analyses showed that strong TFAP2B positivity and pN stage were independent prognostic factors for NSCLC: Hazard ratio (HR), 2.349; 95% CI, 1.411-3.910; *P* = 0.001. Click here for file
