# Supplementary material for: TFAP2B overexpression contributes to tumor growth and a poor prognosis of human lung adenocarcinoma through modulation of ERK and VEGF/PEDF signaling
Source: Mol Cancer. 2014 Apr 26;13:89. doi: 10.1186/1476-4598-13-89 (PMC4021500; doi:10.1186/1476-4598-13-89)
Supplement: Additional file 1: Table S1 — Association of TFAP2B expression with patient’s clinicopathological features in lung adenocarcinomas. Of the 147 lung adenocarcinoma samples, TFAP2B stained strongly in 66 cases (44.9%, 2+ score), staining weakly stained or not at all in 81 cases (55.1%, 1+ or 0 score). There was no statistically significant relationship between TFAP2B expression and gender (P = 0.322), age (P = 0.141), pT stage (P = 0.512), and pN stage (P = 0.060). Table S2. Cox proportional hazards model analysis of prognostic factors in patients with lung adenocarcinomas. Univariate analysis was used to evaluate associations between the patient prognosis and several clinicopathologic factors, including TFAP2B expression (score of 2+ vs. 0, 1+), gender (male vs. female), age (≥ 60 vs. < 60 years), pT stage (tumor size, T3-T4 vs. T1-T2), and pN stage (lymph node metastasis, N1–N2-N3–N4 vs. N0). All of these parameters except gender and age were significantly associated with a poor prognosis. Multivariate Cox proportional hazards regression analyses showed that strong TFAP2B positivity and pN stage were independent prognostic factors for NSCLC: Hazard ratio (HR), 2.349; 95% CI, 1.411-3.910; P = 0.001. [file 1476-4598-13-89-S1.doc]

**Additional file 1: Table S1.** Association of TFAP2B expression with patient’s clinicopathological features in lung ADC

|  | Total  (*n*=147) | TFAP2B low  expression (*n*=81) | TFAP2B high  expression (*n*=66) | *p* |
| --- | --- | --- | --- | --- |
| Gender |  |  |  |  |
| Male | 78 | 40 | 38 | 0.322 |
| Female | 69 | 41 | 28 |
| Age,y |  |  |  |  |
| ≥60 | 77 | 38 | 39 | 0.141 |
| <60 | 70 | 43 | 27 |  |
| pT factor |  |  |  |  |
| T1+T2 | 115 | 65 | 50 | 0.512 |
| T3+T4 | 32 | 16 | 16 |  |
| pN factor |  |  |  |  |
| N0 | 75 | 47 | 28 | 0.060 |
| N1+N2+N3 | 72 | 34 | 38 |  |

Abbreviations: ADC, adenocarcinoma;

**Additional file 1: Table S2.** Cox proportional hazards model analysis of prognostic factors in patients with lung ADC

|  | **HR** | **95% CI** | **Unfavorable/Favorable** | ***p*** |
| --- | --- | --- | --- | --- |
| Univariate analysis |  |  |  |  |
| TFAP2B | 2.446 | 1.473-4.063 | High/low | 0.001^a^ |
| Gender | 1.272 | 0.773-2.094 | Male/female | 0.344 |
| Age,y | 1.149 | 0.697-1.897 | ≥60/<60 | 0.586 |
| pT factor | 2.574 | 1.513-4.380 | T3+T4/T1+T2 | <0.0001^a^ |
| pN factor | 4.033 | 2.314-7.027 | N1+N2+N3/N0 | <0.0001^a^ |
| Multivariate analysis |  |  |  |  |
| TFAP2B | 2.349 | 1.411-3.910 | High/low | 0.001^a^ |
| pT factor | 1.537 | 0.878-2.690 | T3+T4/T1+T2 | 0.133 |
| pN factor | 3.398 | 1.885-6.125 | N1+N2+N3/N0 | <0.0001^a^ |

Abbreviations: ADC, adenocarcinoma; HR, hazard ratio; CI, confidence interval; ^a^*P* < 0.05
